# Noninvasive model for predicting future ischemic strokes in patients with silent lacunar infarction using radiomics

**DOI:** 10.1186/s12880-020-00470-7

**Published:** 2020-07-08

**Authors:** Jie-hua Su, Ling-wei Meng, Di Dong, Wen-yan Zhuo, Jian-ming Wang, Li-bin Liu, Yi Qin, Ye Tian, Jie Tian, Zhao-hui Li

**Affiliations:** 1 Department of Neurology, Zhuhai Hospital Affiliated with Jinan University, No. 79 Kangning Road, Zhuhai, 519000 Guangdong China; 2 School of Artificial Intelligence, University of Chinese Academy of Sciences, Beijing, 100080 China; 3 CAS Key Laboratory of Molecular Imaging, Institute of Automation, Chinese Academy of Sciences, No. 95 Zhongguancun East Road, Beijing, 100190 China; 4 Department of Radiology, Zhuhai People’s Hospital, Zhuhai, 519000 Guangdong China; 5 Department of Orthopedics, Zhuhai Hospital Affiliated with Jinan University, Zhuhai, 519000 Guangdong China; 6 Beijing Advanced Innovation Center for Big Data-Based Precision Medicine, School of Medicine and Engineering, Beihang University, Beijing, 100191 China; 7 Engineering Research Center of Molecular and Neuro Imaging of Ministry of Education, School of Life Science and Technology, Xidian University, Xi’an, 710126 Shaanxi China; 8 Key Laboratory of Big Data-Based Precision Medicine (Beihang University), Ministry of Industry and Information Technology, Beijing, 100191 China

**Keywords:** Stroke, Infarction, Radiomics, Tomography, X-ray computed

## Abstract

**Background:**

This study aimed to investigate integrating radiomics with clinical factors in cranial computed tomography (CT) to predict ischemic strokes in patients with silent lacunar infarction (SLI).

**Methods:**

Radiomic features were extracted from baseline cranial CT images of patients with SLI. A least absolute shrinkage and selection operator (LASSO)–Cox regression analysis was used to select significant prognostic factors based on Model^C^ with clinical factors, Model^R^ with radiomic features, and Model^CR^ with both factors. The Kaplan–Meier method was used to compare stroke-free survival probabilities. A nomogram and a calibration curve were used for further evaluation.

**Results:**

Radiomic signature (*p* < 0.01), age (*p* = 0.09), dyslipidemia (*p* = 0.03), and multiple infarctions (*p* = 0.02) were independently associated with future ischemic strokes. Model^CR^ had the best accuracy with 6-, 12-, and 18-month areas under the curve of 0.84, 0.81, and 0.79 for the training cohort and 0.79, 0.88, and 0.75 for the validation cohort, respectively. Patients with a Model^CR^ score < 0.17 had higher probabilities of stroke-free survival. The prognostic nomogram and calibration curves of the training and validation cohorts showed acceptable discrimination and calibration capabilities (concordance index [95% confidence interval]: 0.7864 [0.70–0.86]; 0.7140 [0.59–0.83], respectively).

**Conclusions:**

Radiomic analysis based on baseline CT images may provide a novel approach for predicting future ischemic strokes in patients with SLI. Older patients and those with dyslipidemia or multiple infarctions are at higher risk for ischemic stroke and require close monitoring and intensive intervention.

## Background

Advances in imaging technologies and in the popularity for image acquisition and post-processing have facilitated radiologic examinations that are universally used to diagnose and monitor cerebrovascular diseases. However, cranial computed tomography (CT) is routinely used as the first-line imaging method for stroke as it is widely available and time- and cost-efficient [1]. Notably, a significant number of patients with lacunar infarction (LI) who underwent CT to evaluate headache, trauma, or limb numbness were diagnosed silent lacunar infarction (SLI), which occurs in individuals without a history of acute neurological dysfunction attributable to a lesion. However, despite being clinically silent, SLI is not a rare event, especially in the aging population [2, 3]. Epidemiologic evidence has shown that, today, the presence of such an event is associated with a twofold increased risk of future stroke [4]. Current circumstances are plagued by over-treatment and over-diagnosis, which may increase potential anxiety and economic burden for the patient [5]. However, remaining unnoticed leads to a blindness of the risk of subsequent stroke and dementia [4, 6]. Although it is recommended that prevention strategies [3] adhere to guidelines of the American Heart Association/American Stroke Association [7], these guidelines appear to lack specialized strategies for high-risk patients. It is unclear whether all individuals with SLI should be considered at equivalent risk as those with symptomatic stroke and should therefore receive antiplatelet drug therapy, statins, or revascularization therapy. However, if a high-risk of ischemic stroke can be detected, the patient may be classified into a future stroke prevention category, and reasonable stroke prevention therapies would be administered.

The relation between SLI and future stroke risk has also been investigated in previous studies under the specific circumstances of increased heart and cerebrovascular risk. Past studies have shown that high-risk factors such as aging, dyslipidemia, hypertension, diabetes mellitus, and carotid artery disease appear to be associated with higher risk of cardiovascular and cerebrovascular disease in patients with SLI [7–14]. However, it has remained unclear whether clinical factors or radiomic features have predictive abilities regarding future strokes. Past studies have emphasized that SLI should not be understood as separable neuroanatomical substrates, and it would probably be subjective and unreasonable to predict future stroke based only on traditional risk factors [15, 16]. Rather than searching for putative markers of future stroke, the combined value of clinical factors and neuroimaging information for predicting future risk is needed. Therefore, noninvasive and effective methods are essential in identifying the risk of future stroke in SLI individuals. Radiomic analysis is an emerging computational tool that exploits copious quantitative features of medical images, providing detailed quantitative information regarding imaging markers that can be applied to modern precision medicine [17, 18]. Briefly, radiomics comprises several procedures. First, radiologists and experts segment the regions of interests (ROI) on medical images. Then, based on the ROIs, the radiomic features, which include the intensity, shape, and texture, are extracted. The most significant features are screened out to support the construction of the prediction model. Radiomics has become increasingly important in cancer diagnosis and treatment [19, 20]. Moreover, studies have indicated that radiomics can be used in the diagnosis and prognosis of neurological diseases such as acute ischemic stroke [21, 22] and multiple sclerosis [23]. These studies have shown that radiomics may become a promising tool for predicting future strokes in patients with SLI.

We hypothesize that radiomic features of the brain reflect the heterogeneity of SLI by identifying low- and high-risk classes and therefore may be able to predict future stroke accurately. By identifying high-risk patients, a more reasonable preventive therapy may be designed to perform intervention, prevent future stroke, and avoid unnecessary costs.

## Methods

### Patients and definition

This retrospective study was approved by the the medical ethics committee of Zhuhai Hospital Affiliated with Jinan University. Because only medical records and radiologic images were reviewed, the requirement for informed consent was waived. All patient records and information were anonymized prior to the analysis. We used a case-cohort design [24] and included patients who visited our hospital between February 2013 and December 2016. From the view of comparability, the ratio of patients with and without stroke of SLI during the follow-up was set as 1:1, such that a cohort member without stroke of SLI at the period of follow-up of their corresponding case was selected at the ratio of 1:1, matched by similar clinical situations. Patients may have arrived at the hospital presenting with clinical symptoms or signs such as headache, dizziness, weakness, numbness of limbs, or other abnormalities. Given that CT is the most common imaging technique in everyday use, and individuals with SLI are often asymptomatic or present with nonspecific neurological symptoms, the application of CT for cerebrovascular disease screening is the common in most developing countries [5]. As instructed, the patients underwent CT to determine the existence of stroke. A total of 2256 participants were included in this study; 148 patients were selected for further analysis (Additional file 1). The inclusion and exclusion criteria are presented in Additional file 2.

We defined the diameter of LI to be between 3 and 20 mm [25]. LI was verified by CT because it is the first-line imaging method used to detect the development of cerebrovascular disease [1, 26–28]. LI is classified as silent if the patient does not have stroke-like symptoms resulting from specific lesions [29] but may have trauma, headache, dizziness, or other symptoms. SLI was diagnosed in patients who lacked a history of transient ischemic attack or stroke-like symptoms according to self-reports, medical records, and radiologic images [30]. To avoid bias, all selected patients did not have ischemic stroke at baseline to ensure that the observed SLIs were truly silent.

### Follow-up examinations and outcomes

Patients received follow-up examinations every 3, 6, and 12 month until the completion of the study (Additional file 3). The minimum follow-up duration was 1 year after baseline CT or until the development of an ischemic stroke (whichever occurred first). Telephone follow-up survey or periodic re-examination were conducted so that clinical events could be recorded. Cranial CT or MRI imaging and routine laboratory tests were performed if a patient experienced stroke-like syndromes or if a recent stroke was suspected. Ischemic stroke was considered the only endpoint for this study. The definition of ischemic stroke was obtained from the guidelines of the American Heart Association/American Stroke Association [30]. At the end of the follow-up period, ischemic stroke was defined as a sudden-onset cerebrovascular event lasting > 24 h that clearly resulted in a new neurological deficit or an increase in an existing deficit. Additionally, evidence of a recent infarct on a reviewed cranial CT or MRI resulting in neurological dysfunction was required in lieu of a self-reported history.

### Clinical factors

Clinical factors gathered from patients included age, sex, number of lesions, routine laboratory tests, carotid artery ultrasound, cardiovascular risk factors, and medical intervention history, as antithrombotic therapies and statin strategies were used regularly during the follow-up period. Cardiovascular risk factors included current smoking status, alcohol abuse, hypertension, diabetes mellitus, and dyslipidemia. Carotid artery ultrasound findings were classified as being either with or without stenosis and plaque [31].

### CT examination

All cranial CT images were derived from two scanners: SOMATOM Definition and SOMATOM Sensation 16 (Siemens Healthcare, Erlangen, Germany). The scan parameters of the SOMATOM Definition were as follows: 120 kV; 200 mAs; 0.28 s rotation time; detector collimation, 128 × 0.6 mm; field of view, 360 × 360 mm; matrix, 512 × 512. The scan parameters of the SOMATOM Sensation 16 were as follows: 120 kV; 250 mAs; 0.42 s rotation time; detector collimation, 64 × 0.6 mm; matrix, 512 × 512.

### Extraction of Radiomic features

ROIs covering the LI were defined as follows: the whole hypodensity area consisting of round or ovoid lesions measuring 3 to 20 mm in the cerebral hemispheric white matter, basal ganglia, or brain stem. ROI size depended on the radii and depths of lacunar lesions reflected in cranial CT images. In cases of multiple lesions, the target lesions were selected based on their density (those with a hypodensity had better recognition) and suitability for accurate repeat measurements. All slices were used for segmentation and were manually drawn by two neuroradiologists. We then loaded the patients’ cranial CT images and the corresponding ROIs into the radiomic feature extraction software PyRadiomics, which was implemented using Python version 3.6.4 (Python, Wilmington, DE, USA; https://www.python.org/) [32]. With original images and filtered images, a large panel of radiomic features quantifying the phenotypic characteristics of medical imaging was extracted according to the methods of previous studies [32]. Five classes of features included shape-based features, intensity-based first-order features, gray-level co-occurrence matrix (GLCM) features, gray-level run-length matrix (GLRLM) features, and gray-level size zone matrix (GLSZM) features. Among these, GLCM, GLRLM, and GLSZM features were descriptors of CT textures. The detailed feature extraction methodology and explanations are described in Additional file 4.

We assessed the reproducibility and stability of radiomic features using an interclass consistency test. Specifically, we randomly selected 30 patients from the 148 enrolled patients (20%), on whose CT images the radiologist had performed the ROI segmentation again. We then calculated the interclass correlation coefficient (ICC) between the corresponding feature pairs respectively derived from the original ROIs and those from the second segmentations [33, 34]. Furthermore, features with ICC > 0.75, which were considered robust and credible, were preserved.

### Feature selection and signature building

With the use of random, computer-generated numbers, patients were divided into training and validation cohorts at a 1:1 ratio. The feature selection procedure was based on the stability, inter-feature redundancy, and lesion-based discriminability of each feature. First, we standardized each feature using the mean and standard deviation derived from the training cohort. Then, we performed a least absolute shrinkage and selection operator (LASSO)–Cox regression analysis to compress the magnitudes of the features, which can help to screen out the most significant features and alleviate overfitting [35]. The minimum redundancy maximum relevance (mRMR) method was then used to rank the features [36]. According to the ranking, we implemented the Cox regression and adopted the forward selection method to determine the final features and corresponding coefficients with which we further calculated the radiomic score and built the radiomic signature.

We built three signatures: radiomic, clinical, and integrative signatures. The radiomic signature was built based on the radiomic score (Rad score), calculated using a linear combination of the selected radiomic features weighted by their corresponding coefficients. The coefficients were derived from the Cox regression. The clinical signature was built according to the clinical score, which was calculated using a linear combination of the selected clinical factors weighted by their corresponding coefficients. Clinical factors were selected using univariate and multivariate Cox regression analyses. The integrative signature was built based on the integrative score, which was calculated through a linear combination of the Rad score and selected clinical factors weighted by their corresponding coefficients.

### Model construction

For further comparisons, three Cox proportional hazards models were constructed based on each corresponding signature: clinical (Model^C^), radiomic (Model^R^), or integrative (Model^CR^).

For model comparisons, we measured the concordance index (C-index) of the three models in the training and validation cohorts with 1000 bootstrap replications for each model. These aimed to evaluate the variance of the C-index and obtain a 95% confidence interval. A C-index value of 0.5 implies no discriminatory ability, while a value of 1.0 indicates perfect discrimination [37].

### Statistical analysis

Age and the number of lesions were treated as continuous variables, whereas sex, vascular risk factors, and study endpoint were treated as binary variables. The Mann–Whitney U test was used to evaluate differences in patients’ ages (non-normal distribution), while the χ^2^ test was used to assess the distribution of the other characteristics. Significant clinical factors were selected using univariate and multivariate regression analyses involving Cox models. All processing such as feature extraction and model construction were implemented in our training cohort, and the validation cohort was totally independent from the training procedure.

The probabilities of stroke-free survival were compared using the Kaplan–Meier method. We used a log-rank test to evaluate the differences in Kaplan–Meier curves for the two groups in each model [38]. Stratified analyses were performed to explore the potential association of the selected signature with stroke-free survival within each subgroup of patients with significant clinical factors. Furthermore, a nomogram was built based on the best model, and discrimination and calibration of the nomogram for 6-, 12-, and 18-month values were measured using the concordance index and calibration curves to compare the expected and observed survival probabilities, respectively [39].

All statistical tests performed were two-sided, and *p* < 0.05 was considered statistically significant. The statistical analysis was performed using Python version 3.6.4.

## Results

### Patient characteristics

A total of 148 patients were retrospectively included in this analysis: stroke and stroke-free patients at a 1:1 ratio. Among all patients, the median (range) follow-up duration was 615 (366–822) days; no difference was found between the training and validation cohorts, which had medians of 620 and 603 days and ranges of 365–855 and 371–855 days, respectively (*p* = 0.36, Mann–Whitney U test). No distribution differences arose between the clinical characteristics or follow-up data of the two cohorts (Table 1).

**Table 1:** Statistical analysis of the clinical characteristics of the training and validation data sets

Characteristics	Training cohort (*n* = 74)	Validation cohort (*n* = 74)	*P*-value
Sex			0.49
Male	40 (54.1)	46 (62.2)	
Female	34 (45.9)	28 (37.8)	
Age, years	75 [68–82]	77 [72–82]	0.10
< 76 years	39 (52.7)	28 (37.8)	
≥ 76 years	35 (47.3)	46 (62.2)	
Multiple infarctions			0.61
Yes	31 (41.9)	27 (36.5)	
No	43 (58.1)	47 (63.5)	
Smoking status			0.68
Yes	14 (18.9)	16 (21.6)	
No	60 (81.1)	58 (78.4)	
Alcohol abuse			0.27
Yes	2 (2.7)	6 (8.1)	
No	72 (97.3)	68 (91.9)	
Hypertension			0.84
Yes	57 (77.0)	58 (78.4)	
No	17 (23.0)	16 (21.6)	
Diabetes mellitus			0.99
Yes	28 (37.8)	33 (44.6)	
No	46 (62.2)	41 (55.4)	
Dyslipidemia			0.29
Yes	57 (77.0)	63 (85.1)	
No	17 (23.0)	11 (14.9)	
Internal carotid plaque^a^			0.79
Yes	59 (86.8)	61 (89.7)	
No	9 (13.2)	7 (10.3)	
Internal carotid stenosis^a^			0.36
Yes	4 (5.9)	8 (11.8)	
No	64 (94.1)	60 (88.2)	
Using statin strategies			0.80
Yes	10 (13.5)	9 (12.2)	
No	64 (86.5)	65 (87.8)	
Using antithrombotic therapies			0.77
Yes	11 (7.4)	10 (6.7)	
No	63 (42.6)	64 (43.3)	

Values are presented as n (%) or median (range)

Internal carotid plaque^a^ and internal carotid stenosis were only collected from 136 patients who had initial carotid artery ultrasound reviews

The differences in the patients’ ages were evaluated using the Mann–Whitney U test

The distribution of the other characteristics was assessed using the χ^2^ test

### Radiomic features

**Fig. 1:**
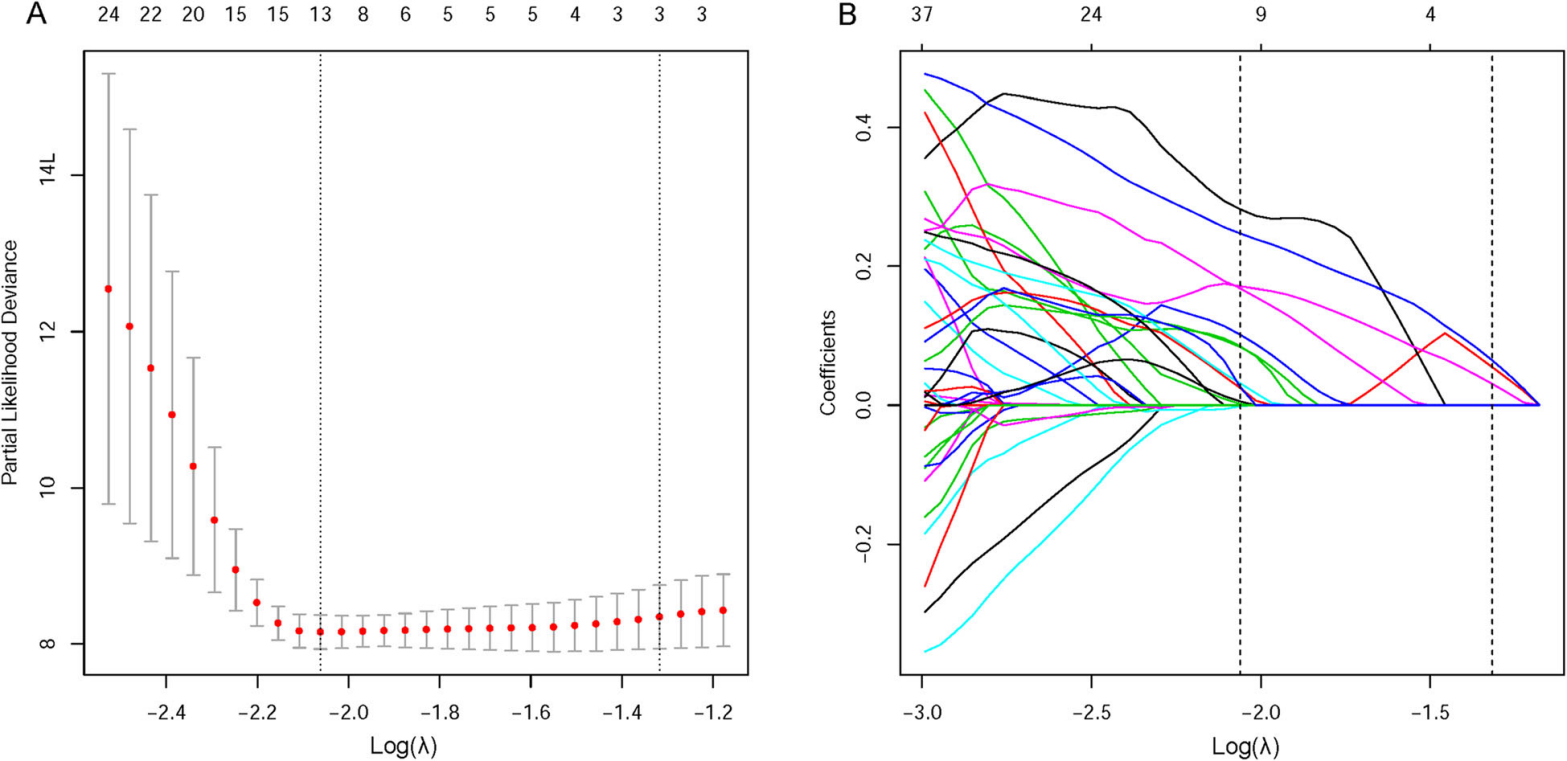
Feature selection using the LASSO–Cox regression model. (**a**) Tuning parameter λ selection in the LASSO–Cox regression model. Partial likelihood deviance was plotted against the log (λ) sequence. Error bars represent 95% CIs. Identification of the optimal penalization coefficient λ in the LASSO model used tenfold cross-validation and minimum criterion. As a result, a λ value of 0.127 with log (λ) = − 2.061 was selected. The dotted vertical line was plotted at the selected value using tenfold cross-validation, for which the optimal λ resulted in eight, nonzero coefficients. The numbers on the top axis represent the quantity of the features. (**b**) LASSO coefficient profiles of 1209 features (represented by different-colored curves) selected using univariate Cox regression analysis were plotted against the log (λ) sequence. CI, confidence interval; LASSO, least absolute shrinkage and selection operator

Of the 1209 features extracted from CT images, 844 were confirmed to be robust by the interclass consistency test (ICC, 0.75–0.99). Of these, 13 features were selected using the LASSO algorithm. The process of feature selection is shown in Fig. 1. After forward selection, four features (wavelet-LLH_glszm_SizeZoneNonUniformity, squareroot_firstorder_Maximum, wavelet-LHL_firstorder_Skewness, and logarithm_glcm_Idn) were finally selected to construct the radiomic signature. Details and implications of these four features are provided in Additional file 5.

### Signature building and model construction

**Fig. 2:**
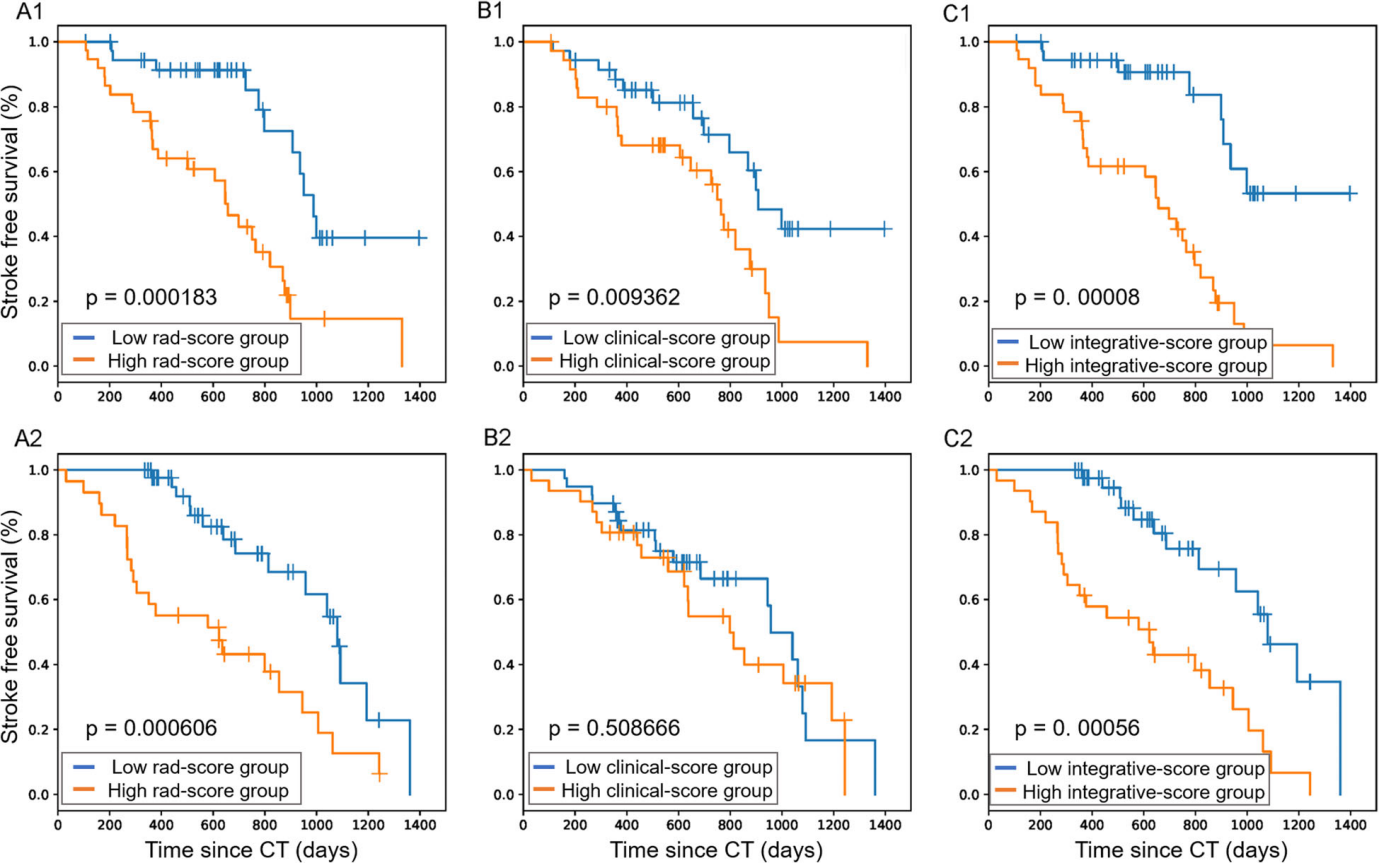
Kaplan–Meier curves of the training (**a1**, **b1**, and **c1**) and validation (**a2**, **b2**, and **c2**) cohorts, with patients divided into low- and high-risk groups, respectively. These were based on the median Rad score (**a1** and **a2**) of 0.46, median clinical score (B1 and B2) of 0.08, and median integrative score (**c1** and **c2**) of 0.17. Small vertical tick marks on the plot indicate individual patients whose survival probabilities have been right censored. A higher score represents higher risk. **a1** and **a2** curves suggest that the radiomic signature is significantly associated with stroke-free survival in both cohorts. For **b1** and **b2**, the clinical signature had a significant association with stroke-free survival only in the training cohort but showed poor separation performance in the validation cohort. Regarding **c1** and **c2**, the integrative signature had a more significant association with stroke-free survival than the other two signatures for both training and validation cohorts. Rad score, radiomic score

**Fig. 3:**
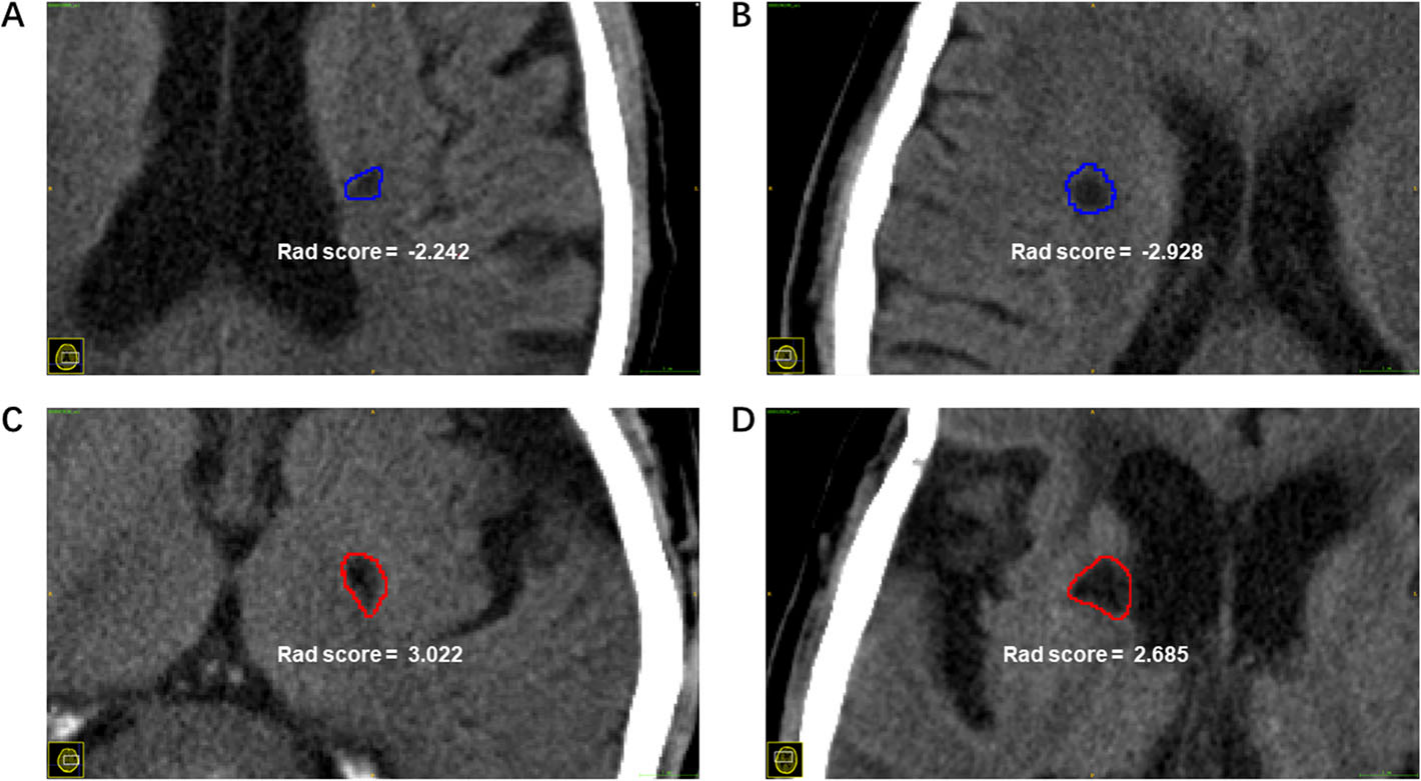
CT images of four patients and corresponding ROIs were sampled from the whole cohort. (**a**) and (**b**) indicate stroke-free patients, whereas (**c**) and (**d**) indicate patients who suffered future strokes. Although the Rad scores vary considerably within the two groups, they are nearly indistinguishable to the naked eye. This suggests that Rad scores, as derived from selected radiomic features, quantitatively represent the latent heterogeneity of ROIs and further predict risk of future strokes. The above images were resized to a scale of 15 voxels/mm, and the intensity range was windowed by the interval [0, 100] for good visual effect. CT, computed tomography; ROI, region of interest

A multivariate logistic regression analysis identified the radiomic signature (hazard ratio [HR], 2.31; 95% confidence interval [CI], 1.59–3.35), dyslipidemia (HR, 4.91; 95% CI, 1.41–17.19), age (HR, 1.03; 95% CI, 0.99–1.09), and number of lesions (HR, 1.03; 95% CI, 0.98–1.08) as independent predictors. These were used to construct the signatures and models. The radiomic, clinical, and integrative signatures were respectively built based on the Rad, clinical, and integrative scores calculated for each patient. The coefficients of each selected significant factor for the corresponding calculation and formulas of the three scores are shown in Additional file 6. To assess differences in stroke-free survival (Fig. 2), we selected the medians of the Rad and integrative score series as the thresholds (Rad score, 0.46; integrative score, 0.17). The four selected features that were used to obtain the Rad score and construct the radiomic signature described distinctions between the images of stroke and stroke-free patients (Fig. 3). Two of these (wavelet-LLH_glszm_SizeZoneNonUniformity and logarithm_glcm_Idn) slightly emphasized the heterogeneity of the neighboring voxels in the ROI. As the coefficients of all features were positive, a patient with a more heterogenous ROI would be at higher risk. Meanwhile, the other two features were related to higher intensity values (squareroot_firstorder_Maximum) and the asymmetrical distribution of the histogram (wavelet-LHL_firstorder_Skewness), indicating a worse prognosis with an ROI containing a higher overall intensity level. Figure 3 demonstrates the aforementioned interpretations regarding these features. Detailed implications of the four selected features are illuminated in Additional file 5.

**Fig. 4:**
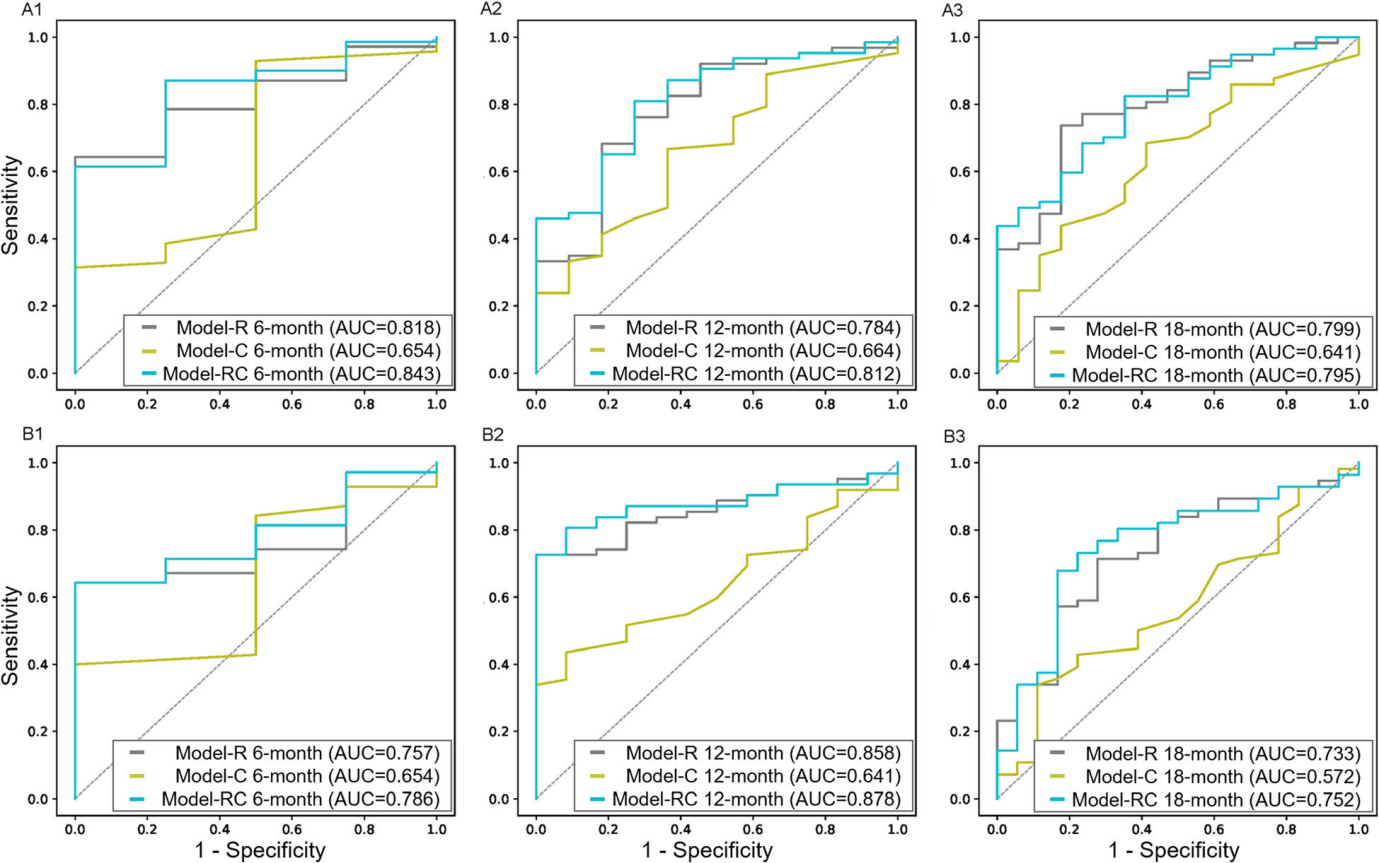
Six-, 12-, and 18-month ROC curves of Model^R^, Model^C^, and Model^CR^ in the training cohort (**a1, a2**, and **a3**) and validation cohort (**b1, b2**, and **b3**). The graphs suggest that Model^CR^ can more accurately predict SLI. Model^C^, model based solely on clinical factors; Model^R^, model based only on radiomic features; Model^CR^, model based on both clinical and radiomic factors; SLI, silent lacunar infarction; ROC, receiver operating characteristic

Patients with Rad scores < 0.46 and integrative scores < 0.17 were more likely to survive a future stroke. The clinical signature had a significant association with stroke-free survival only in the training cohort but showed poor separation performance in the validation cohort (Fig. 2). For the training cohort, the C-indices (95% CI) of Model^C^, Model^R^, and Model^CR^ were 0.6617 (0.57–0.75), 0.7734 (0.69–0.84), and 0.7864 (0.70–0.86), respectively, and for the validation cohort, they were 0.6911 (0.57–0.81), 0.7066 (0.60–0.81), and 0.7140 (0.59–0.83), respectively. According to the receiver operating characteristic (ROC) curves (Fig. 4), Model^CR^ had the best performance, with 6-, 12-, and 18-month areas under the curve (AUCs) of 0.84, 0.81, and 0.79 for the training cohort and 0.79, 0.88, and 0.75 for the validation cohort, respectively (Table 2).

**Table 2:** Comparisons of the areas under the curve of the three prognostic models

	Model^R^	Model^C^	Model^CR^
Training cohort			
6 months	0.818	0.653	0.843
12 months	0.784	0.663	0.812
18 months	0.799	0.641	0.795
Validation cohort			
6 months	0.757	0.654	0.786
12 months	0.858	0.641	0.878
18 months	0.733	0.572	0.752

*Model*^*R*^ model consisting of the radiomic signature, *Model*^*C*^ model consisting of clinical factors, *Model*^*CR*^ model consisting of the clinical signature and radiomic signature

### Prognostic Nomogram

**Fig. 5:**
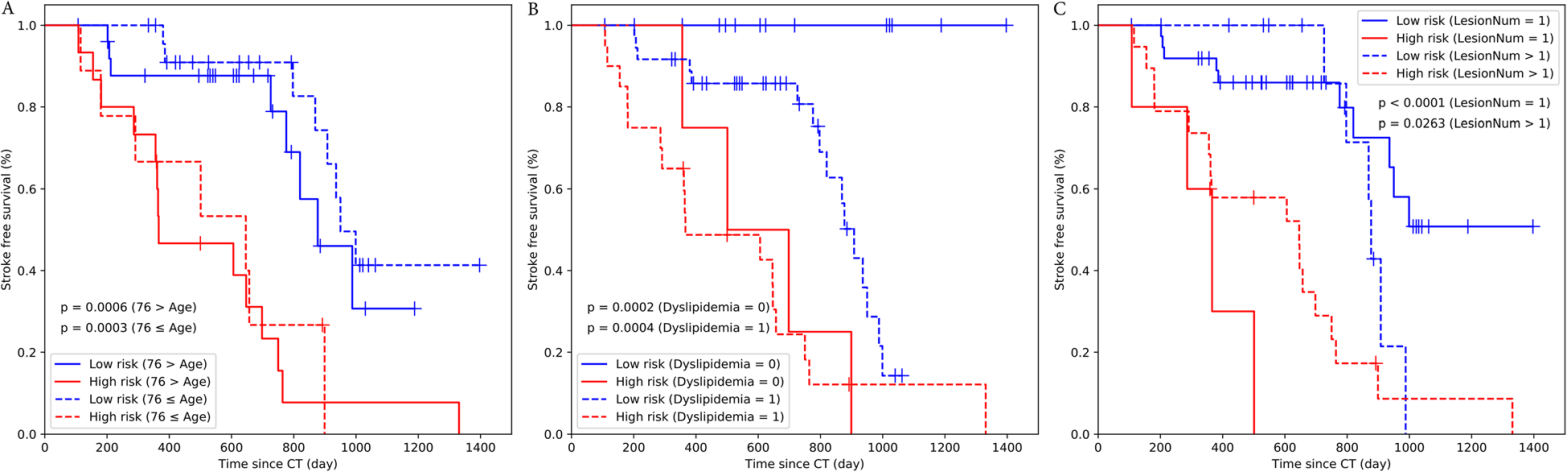
Stratified analyses were performed to evaluate stroke-free survival in three significant clinical characteristic subgroups: (**a**) age, (**b**) dyslipidemia, and (**c**) the number of lesions. To achieve this, Kaplan–Meier survival curves of the low- and high-risk groups were compared. Small vertical tick marks indicate individual patients whose survival probabilities have been right censored

**Fig. 6:**
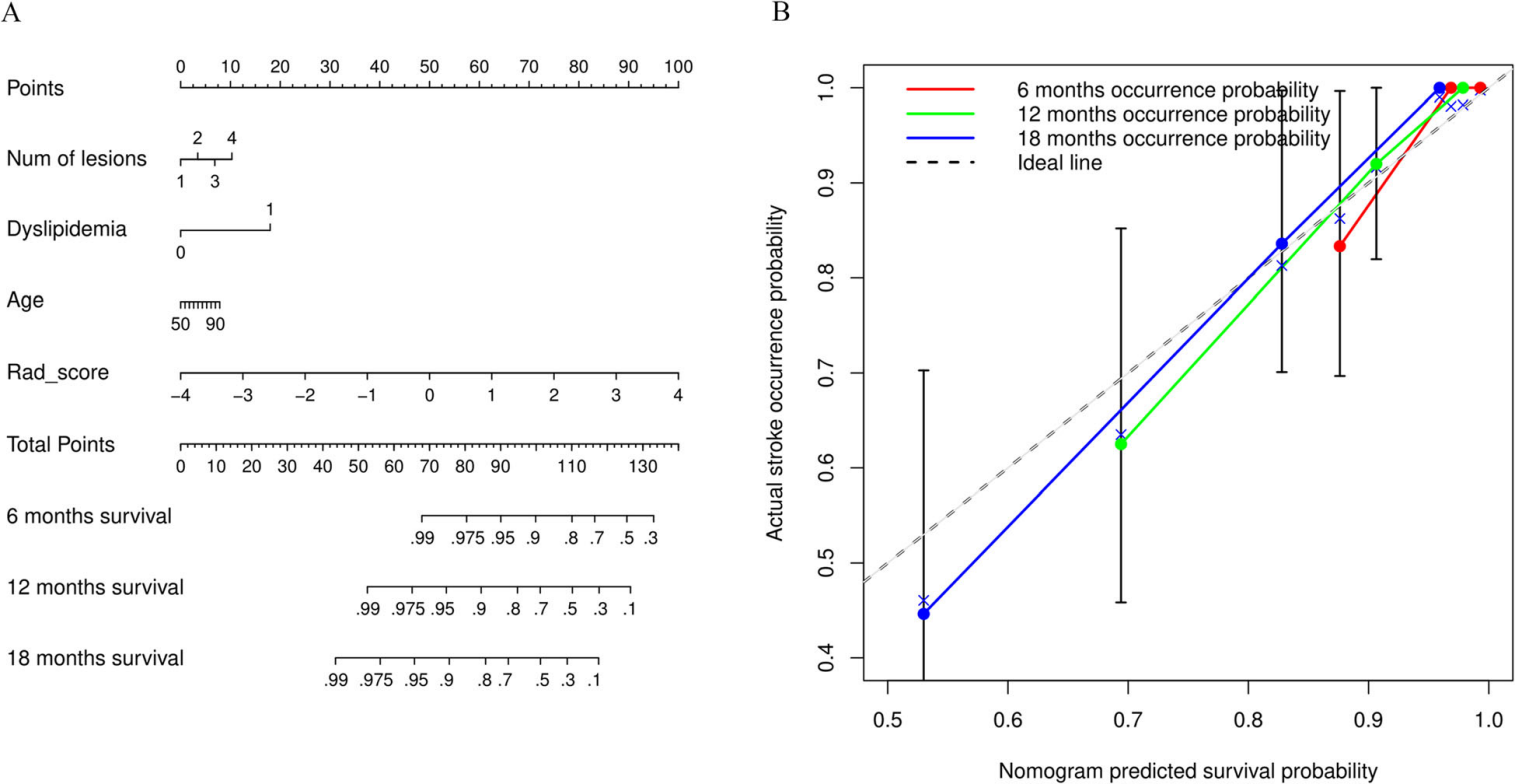
Prognostic nomogram for radiomic, clinical, and integrative scores. (**a**) Prognostic nomogram. The patient’s integrative score, calculated based on the integrative signature of the integrative score axis, was identified. A line was then drawn upward toward the point axis to determine the number of points the patient receives for the integrative score. After repeating the process for the dyslipidemia and age axes, the points achieved for each of the three factors were added. Finally, the total sum of the points on the total points axis was determined, and a line was subsequently drawn straight down to derive the patient’s overall probability of 6-, 12-, and 18-month survival. These points depict the probability of ischemic stroke. (**b**) Calibration curves of the prognostic nomogram. The curves suggest an acceptable calibration performance for the predicted (*x*-axis) and actual 6-, 12-, and 18-month stroke occurrence probabilities (*y*-axis). The diagonal dotted line represents an ideal estimation. This shows a good agreement between estimation and actual observation

Stratified analyses (Fig. 5) showed an association between the integrative signature and stroke-free survival in all subgroups. Regarding age (Fig. 5a), the integrative signature had a significantly stratified younger group (< 76 years) and older group (≥ 76 years), which were categorized as low- and high-risk groups, respectively (log-rank test, *p* = 0.0006 vs. *p* = 0.0003, respectively). The integrative signature divided the subjects into low- and high-risk groups with respect to the existence of dyslipidemia or multiple infarctions (χ^2^ test: *p* = 0.0002 vs. *p* = 0.0004; *p* < 0.0001 vs. *p* = 0.0263, respectively) (Fig. 5b, c). A prognostic nomogram was constructed based on Model^CR^ (Fig. 6a). The calibration curves of the nomogram for the probability of future stroke at 6-, 12-, and 18- months are shown in Fig. 6b.

## Discussion

Herein, we aimed to explore a potential approach of predicting prognosis in patients with SLI. The results of this study indicated that radiomic features had the ability to distinguish SLI patients with a high-risk of future stroke. Moreover, our study showed that the Model^CR^, which combined clinical factors and radiomic features, had the best performance in predicting stroke-free survival. Therefore, the model may aid in predicting the prognosis of patients with SLI.

To date, SLI is significantly associated with an increased risk of symptomatic stroke [4], but it is mainly used as a subjective and qualitative marker. The radiomics approach adopted in this study is more quantitative and probably feasible for predicting future ischemic stroke in patients with SLI. The selected features reflected the size zone volume heterogeneity, high intensity values, asymmetrical histogram distribution, and image volumes. Given that the coefficients of these features were all positive during model construction, a higher value of any radiomic feature may therefore indicate a worse prognosis.

Increased age is the most widely accepted high-risk factor for symptomatic stroke as it is strongly associated with future stroke in both the presence and absence of SLI [3]. A recent study reported that patients of all ages with multiple silent brain infarcts had a 2.5-fold higher risk of recurrent ischemic stroke [40]. Our results verified these findings and extended the results to comprise patients without stroke at baseline to confine our analysis to truly silent brain infarcts. While we did not verify the association between statin strategies and endpoints, we assumed that dyslipidemia lead to a higher risk of future strokes in patients with SLI according to the report of the trial on Stroke Prevention by Aggressive Reduction in Cholesterol Levels [9]. As these data are limited by the sample size of only 19 patients who regularly used statin strategies, however, this requires further investigation to examine the mechanisms and their associations.

Interestingly, in our study, the model built by clinical factors showed relatively poor performance in the C-index and AUC values, and the separation performance in Kaplan–Meier curves was inferior to that of other models. Meanwhile, the C-indices of Model^CR^ in the training and validation cohorts were the best among the three models. This provides preliminary evidence that the radiomic signature is a potential predictor, whereas clinical parameters make only limited contributions to the Cox model. Not coincidentally, radiomic features seemed to be the most powerful predictor in the radiomic-based model based on integrative factors for the application of cancers such as in non-small cell lung cancer and Glioblastoma [41, 42]. However, it remains difficult to elaborate the physiological interpretation of such a phenomenon immediately. Norrving et al. reported that SLI was a much more active and dynamic process than the simplistic commonly held paradigm of risk factors corresponding to specific infarct, and then triggering negative outcomes [15]. Thus, precise management of SLI solely on the basis of known clinical risk factors is challenging. Radiomic features were believed to provide quantitative information of tissue phenotype, which is complementary to the other diagnostic schemes [43]. It is therefore more likely to be used in various tasks in SLI such as risk assessment, detection, and prognosis.

This study has several limitations. Its retrospective design precluded investigation of some factors [29] such as chronic kidney disease, hyperhomocysteinemia, and detailed medical interventions. Despite the strong relationship between SLI and future stroke, as with atrial fibrillation (the most common cause of cardioembolism), data relating SLI to subsequent risks were limited [44]. For instance, we considered large-artery atherosclerosis to be a major cause of cerebral infarction and thus excluded patients with cardioembolic stroke to avoid uncertainty, which may have introduced selection bias. In addition, although previous studies focused primarily on long-term follow-up examinations, older subjects who enrolled in our study may experience stroke on a short-term timeline. Therefore, only short-term prognoses were investigated. We also used only CT-derived radiomic features, as CT is usually the first-line method to detect cerebrovascular diseases. However, MRI is a more sensitive tool in the diagnosis of LI and may better facilitate appropriate medical decisions. Furthermore, it may have introduced selection bias in the statistical analyses for the incidence of stroke, as we wanted to ensure that the patients with SLI were truly silent. The conclusions drawn herein may therefore be made more robust after future tests with a larger sample size, multi-center testing, and various imaging modalities.

## Conclusion

The results of our study imply that a noninvasive and convenient radiomic-based model may facilitate the management of incidentally found SLI. This therefore has the potential to formulate intensive preventive measures for the reduction of stroke risk. A radiomic approach based on cranial CT images may help identify the high-risk patients. Our model suggests that the risk of stroke is higher in older patients and patients with dyslipidemia or multiple infarctions, indicating that intensive measures for these patients are preferred. Further studies should be conducted to improve the accuracy of predicting ischemic stroke via imaging modalities and radiomics analysis.

## Supplementary information

**Additional file 1.** Flowchart of patient recruitment process. n, number of patients; TIA: Transient Ischemic Attacks

**Additional file 2.** Inclusion and exclusion criteria.

**Additional file 3.** Flowchart for the follow-ups process. CT: Computed tomography; MRI: Magnetic Resonance Imaging

**Additional file 4.** Radiomic feature extraction methodology.

**Additional file 5.** The implications of the 4 chosen features.

**Additional file 6.** Signature score calculations.

## Data Availability

The datasets used and/or analysed during the current study are available from the corresponding author on reasonable request.

## Abbreviations

LI: Lacunar infarction
SLI: Silent lacunar infarction
