# Supplementary material for: Noninvasive model for predicting future ischemic strokes in patients with silent lacunar infarction using radiomics
Source: BMC Med Imaging. 2020 Jul 8;20:77. doi: 10.1186/s12880-020-00470-7 (PMC7346609; doi:10.1186/s12880-020-00470-7)

**Additional file 1:** Flowchart of patient recruitment process. n, number of patients; TIA: Transient Ischemic Attacks


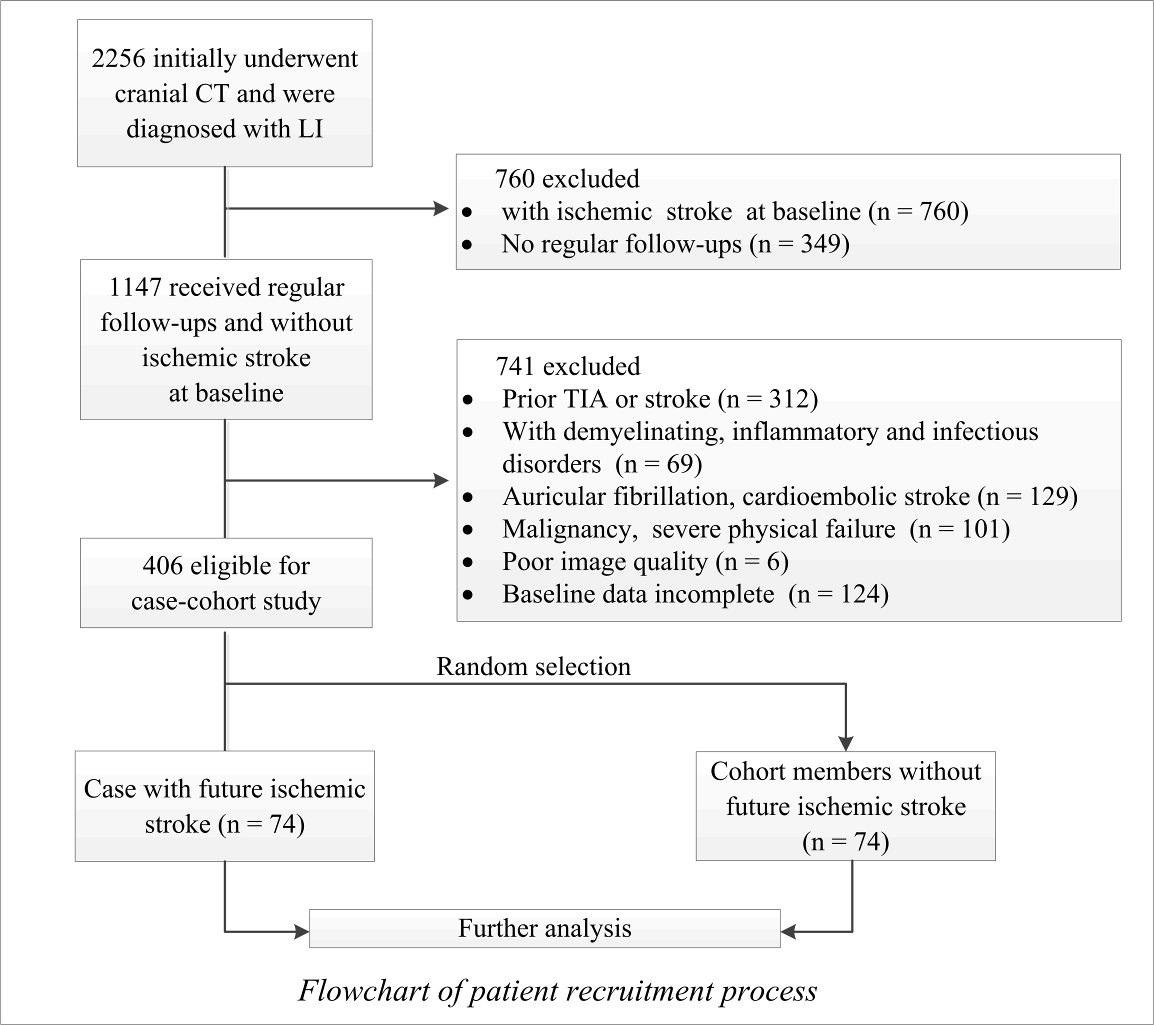

Supplement: Supplementary file 1 — Additional file 1. Flowchart of patient recruitment process. n, number of patients; TIA: Transient Ischemic Attacks [file 12880_2020_470_MOESM1_ESM.docx]
