# Supplementary material for: Noninvasive model for predicting future ischemic strokes in patients with silent lacunar infarction using radiomics
Source: BMC Med Imaging. 2020 Jul 8;20:77. doi: 10.1186/s12880-020-00470-7 (PMC7346609; doi:10.1186/s12880-020-00470-7)
Supplement: Supplementary file 2 — Additional file 2. Inclusion and exclusion criteria. [file 12880_2020_470_MOESM2_ESM.docx]

**Additional file 2:** Inclusion and exclusion criteria.

Subjects meeting the following criteria were included: (1) risk of stroke during the follow-up (until the end of December 2017) and eventually presence of an ischaemic stroke (those with similar characteristics and those without stroke during the same period were compared); (2) cranial CT and diagnosis of LI by two experienced neuroradiologists (clinical outcome and pre-existing reports were blinded to neuroradiologists; patients were only enrolled when they reached an agreement regarding the diagnosis of LI); (3) absence of acute ischaemic stroke at baseline; and (4) absence of demyelinating, inflammatory or infectious disorders of the neurological system (for the diagnostic accuracy of SLI). The exclusion criteria were as follows: (1) diagnosis of auricular fibrillation or symptomatic stroke with cardioembolic sources and (2) malignancy or severe physical failures, such as chronic liver disease or heart failure.
