# Supplementary material for: Noninvasive model for predicting future ischemic strokes in patients with silent lacunar infarction using radiomics
Source: BMC Med Imaging. 2020 Jul 8;20:77. doi: 10.1186/s12880-020-00470-7 (PMC7346609; doi:10.1186/s12880-020-00470-7)

**Additional file 3:** Flowchart for the follow-ups process.

CT: Computed tomography; MRI: Magnetic Resonance Imaging


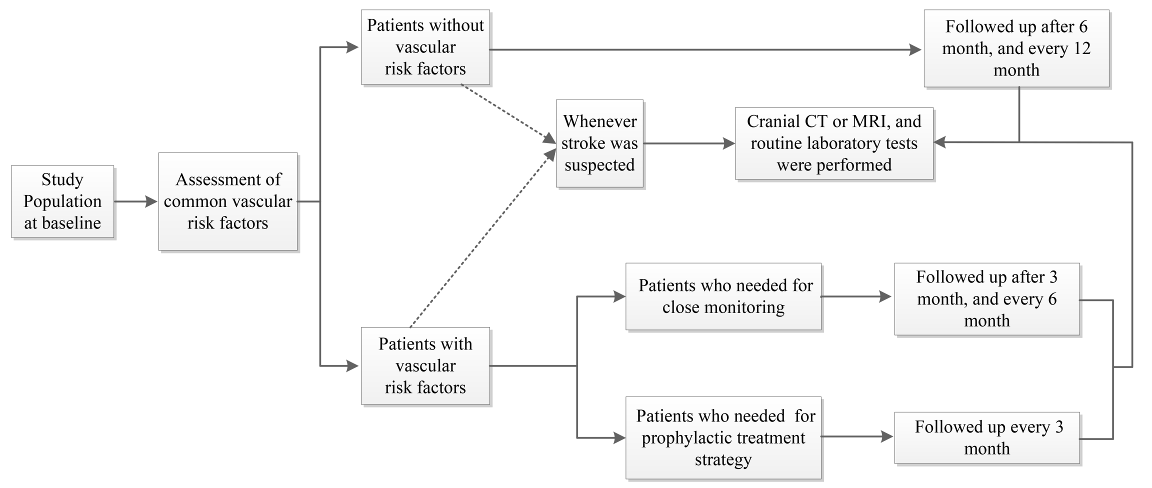

Supplement: Supplementary file 3 — Additional file 3. Flowchart for the follow-ups process. CT: Computed tomography; MRI: Magnetic Resonance Imaging [file 12880_2020_470_MOESM3_ESM.docx]
