# Supplementary material for: Noninvasive model for predicting future ischemic strokes in patients with silent lacunar infarction using radiomics
Source: BMC Med Imaging. 2020 Jul 8;20:77. doi: 10.1186/s12880-020-00470-7 (PMC7346609; doi:10.1186/s12880-020-00470-7)
Supplement: Supplementary file 4 — Additional file 4. Radiomic feature extraction methodology. [file 12880_2020_470_MOESM4_ESM.docx]

**Additional file 4:** Radiomic feature extraction methodology.

To reveal the heterogeneity between patients who had ischaemic stroke and those who did not have ischaemic stroke based on cranial CT images, we loaded the patient’s cranial CT image and corresponding mask image (ROIs) into the radiomic features extraction software (Pyradiomics, which was implemented by Python 3.6.4). In this study, all software was processed on a 64-bit Windows ten computer. On each patient’s original CT image, we applied a few filters to derive different image types that could be used to extract features. The available image types were as follows:

- Original: No filter applied.
- Wavelet: The un-decimated 3-dimensional (3D) wavelet transform was used to decompose the original image, which can be regarded as pre-processing prior to feature extraction. By changing the ratio of a high-frequency signal to a low-frequency signal in images, wavelet transform increases the information of the low-frequency signal. We set *L* and *H* as the low- and high-pass functions, respectively, and *X* as the decomposing image and labelled the wavelet decompositions of *X* as *X*_LLL_, *X*_LLH_, *X*_LHL_, *X*_LHH_, *X*_HLL_, *X*_HLH_, *X*_HHL_ and *X*_HHH_. Then, we obtained eight new images that are decomposed in three directions (x, y and z). The size of each decomposition is equal to the original image, and each decomposition is a shift invariant.
- LoG: Laplacian of the Gaussian filter and edge enhancement filter. This emphasises the areas of changes in the grey level, where sigma defines how coarse the emphasised texture should be. A low sigma places emphasis on fine textures (change over a shorter distance), whereas a high sigma value emphasises coarse textures (change in grey level over a larger distance).
- Square: Takes the square of the image intensities and linearly scales them back to the original range. Negative values in the original image will be reverted to negative after applying the filter.
- SquareRoot: Takes the square root of the absolute image intensities and scales them back to the original range. Negative values in the original image will be reverted to negative after applying the filter.
- Logarithm: Takes the logarithm of the absolute intensity + 1. Values are scaled to the original range, and negative original values are reverted to negative after applying the filter.
- Exponential: Takes the exponential, where the filtered intensity is e^ (absolute intensity). Values are scaled to the original range, and negative original values are reverted to negative after applying the filter.
- Gradient: Returns the magnitude of the local gradient.

A large panel of radiomic features quantifying five kinds of phenotypic characteristics on each medical image type was extracted for each patient in our study. These five feature classes are as follows:

- First-order features: First-order statistics describe the distribution of voxel intensities within the image region defined by the mask through commonly used and basic metrics.
- Shape features: In this group of features, we included descriptors of the 3D size and shape of the ROI. These features are independent from the grey-level intensity distribution in the ROI; therefore, they are only calculated for the non-derived image and mask.
- Grey-level co-occurrence matrix (GLCM) features: GLCM is the matrix function that describes the distance and angle of each pixel. By calculating the correlation between the two grey levels with certain directions and distances, GLCM can reflect integrated information regarding the direction, interval, amplitude and frequency of images. The radiomic features in the GLCM mainly consisted of energy, entropy, correlation, contrast, homogeneity, autocorrelation, mean, variance, dissimilarity and angular second moment.
- Grey-level run length matrix (GLRLM) features: GLRLM quantifies the grey-level runs in an image. A grey-level run is defined as the length (number of consecutive pixels) that has the same grey-level value. The radiomic features of the GLRLM mainly consisted of features such as run length non-uniformity, short/long run emphasis and grey-level non-uniformity.
- Grey-level size zone matrix (GLSZM) features: GLSZM quantifies grey-level zones in an image. A grey-level zone is defined as the number of connected voxels that share the same grey-level intensity. This matrix is particularly efficient to characterise the homogeneity, non-periodicity in texture or speckle-like texture.
