# Supplementary material for: Noninvasive model for predicting future ischemic strokes in patients with silent lacunar infarction using radiomics
Source: BMC Med Imaging. 2020 Jul 8;20:77. doi: 10.1186/s12880-020-00470-7 (PMC7346609; doi:10.1186/s12880-020-00470-7)

**Additional file 5:** The implications of the 4 chosen features.

| Feature Name | Filter | Group | Description |
| --- | --- | --- | --- |
| wavelet-LLH_glszm_  SizeZoneNonUniformity | Wavelet-LLH | GLSZM based features | $SZN=\frac{\sum_{j=1}^{N_{s}} \left( \sum_{i=1}^{N_{g}} P\left( i,j \right) \right)^{2}}{N_{z}}$  Size-zone nonuniformity (SZN) measures the variability of size-zone volumes in the image, with a higher value indicating more heterogeneity in size-zone volumes. |
| squareroot_firstorder_  Maximum | Squareroot | First order statistics | $maximum=max(X)$  The maximum gray-level intensity within the ROI. This tends to emphasize the regions with high intensity values. |
| wavelet-LHL_firstorder_  Skewness | Wavelet-LHL | First order statistics | $skewness=\frac{\mu_{3}}{\sigma^{3}}=\frac{\frac{1}{N_{p}}\sum_{i=1}^{N_{p}} \left( X\left( i \right)-\bar{X} \right)^{3}}{\left( \sqrt{\frac{1}{N_{p}}\sum_{i=1}^{N_{p}} \left( X\left( i \right)-\bar{X} \right)^{2}} \right)^{3}}$  where μ3 is the third central moment.  Skewness measures the asymmetry of the distribution of values of the mean value. Depending on where the tail is elongated and the mass of the distribution is concentrated, this value can be positive or negative. This tends to emphasize the asymmetry distribution of histogram. |
| logarithm_glcm_  Idn | Logarithm | GLCM based features | $IDN=\sum_{i=1}^{N_{g}} \sum_{j=1}^{N_{g}} \frac{p\left( i,j \right)}{1+\left( \frac{\left\vert i-j \right\vert}{N_{g}} \right)}$  Inverse difference normalized (IDN) is a measure of the local heterogeneity of an image. IDN normalizes the difference between the neighboring intensity values by dividing the total number of discrete intensity values, with a higher value indicating more heterogeneity in volumes. |

The feature name was split by an underscore ‘_’, where the first part of the name was the image filter used by the feature, the second part was the class to which the feature belonged and the third part indicated the specific formula of this feature. With regard to wavelet filters, they decomposed original image separately in 3 directions (x, y and z), with either low- or high-pass functions, deriving 8 image filter (wavelet_LLL, _LLH, _LHL, LHH, HLL, HLH, HHL and HHH). In features’ formula, we let **X** denote the 3D image matrix with$N_{p}$voxels, while$\bar{X}$is the mean value of the image matrix. $N_{g}$is the number of non-zero bins. $P(i, j)$ is the dependence matrix, while $p(i, j)$ is the normalised $P(i, j)$*.* The $(i, j)$ element equals the number of zones with grey level$i$ and size$j$ appear in images.


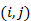

Supplement: Supplementary file 5 — Additional file 5. The implications of the 4 chosen features. [file 12880_2020_470_MOESM5_ESM.docx]
