# Supplementary material for: Noninvasive model for predicting future ischemic strokes in patients with silent lacunar infarction using radiomics
Source: BMC Med Imaging. 2020 Jul 8;20:77. doi: 10.1186/s12880-020-00470-7 (PMC7346609; doi:10.1186/s12880-020-00470-7)
Supplement: Supplementary file 6 — Additional file 6. Signature score calculations. [file 12880_2020_470_MOESM6_ESM.docx]

**Additional file 6:** Signature score calculations.

Rad score = 0.8645 × wavelet-LLH_glszm_SizeZoneNonUniformity

+ 0.8092 × squareroot_firstorder_Maximum

+ 0.4425 × wavelet-LHL_firstorder_Skewness

+ 0.4188 × logarithm_glcm_Idn

Clinical score = 1.0808 × dyslipidaemia + 0.3201 × lesion number + 0.0242 × age

Integrative score = 0.8372 × Rad score + 1.5922 × dyslipidaemia + 0.0325 ×lesion number + 0.0242 × age
